# Substance use in LGBTQ+ populations

**DOI:** 10.1038/s43856-022-00132-5

**Published:** 2022-06-16

## Abstract

Dr. Daniel Demant is an Epidemiologist and Senior Lecturer in Public Health at the University of Technology Sydney and a Visiting Fellow in the School of Public Health and Social Work at the Queensland University of Technology, Brisbane, Australia. His research focuses on health in disadvantaged populations and he has a particular interest in sexual health and substance use in LGBTQ+ populations. In this Q&A, we ask Dr. Demant a series of questions on disparities in substance use in LGBTQ+ people, the potential reasons underlying such disparities and harms associated with them, and the direction of research in this area.

**Figure:**
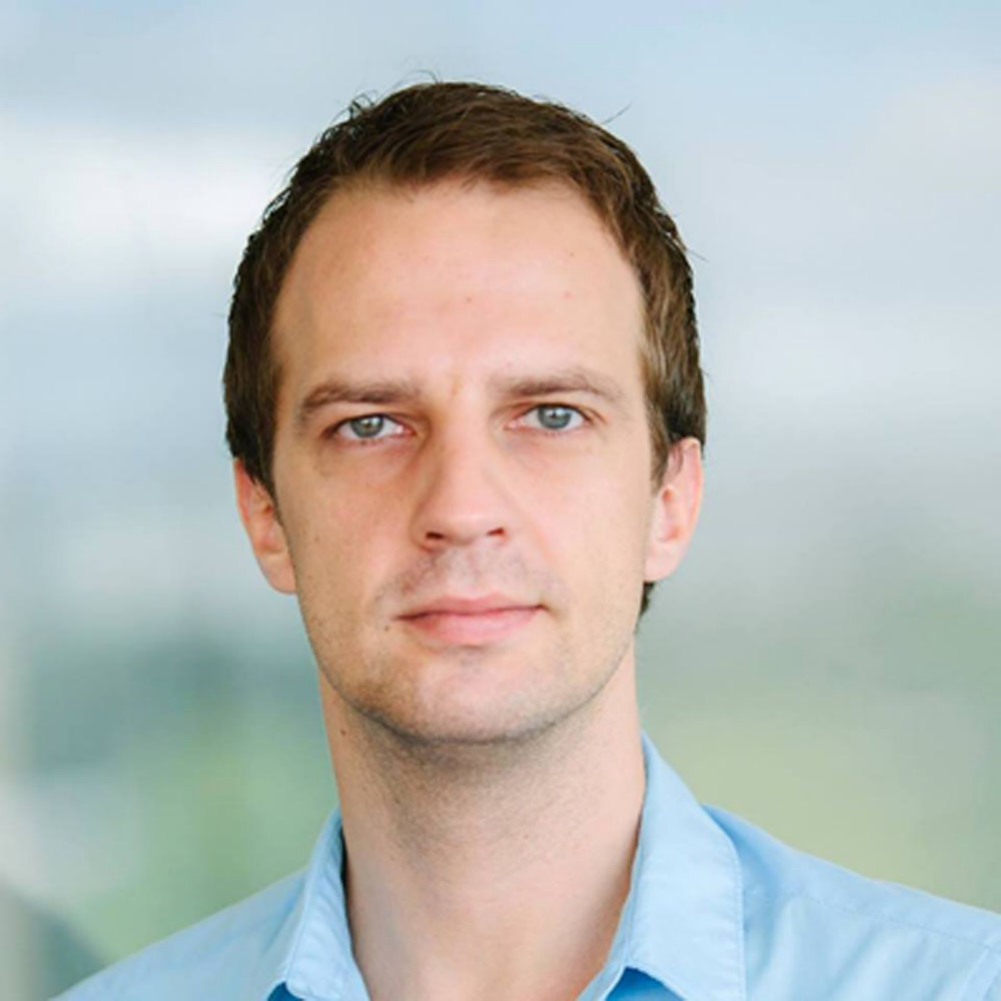
Daniel Demant

What kind of substance use issues affect LGBTQ+ populations?

LGBTQ+ people have worse health outcomes than the general population across a variety of areas including substance use. The literature in the field found disproportionate levels of substance use, including harmful substance use and dependency syndromes. This includes generally higher levels of alcohol use, particularly risky long- and short-term alcohol consumption, such as binge drinking. Similar, there is a higher smoking prevalence and increased use of other drugs – both licit and illicit. Particularly interesting is that research overall suggests that LGBTQ+ communities are not only more likely to use substances but also use them more frequently and start using substances at an earlier age. A more recent phenomenon, particularly in gay and bisexual men, is chemsex, the act of intentionally engaging in sexual activities under the influence of substances such as methamphetamine, mephedrone and GHB that can lead to significant mental and physical harm to some engaged in these activities.

What are the harms – both in terms of physical and mental health – associated with substance use in LGBTQ+ people?

The harms and consequences of substance use do not differ from the harms they cause in other populations in most instances. It is also important to differentiate between substance use and harmful substance use as not all instances of substance use, including so-called ‘hard drugs’, will result in meaningful harm to a person or their community. However, prolonged harmful substance use can result in a range of negative consequences for a person’s physical and mental health. In the case of LGBTQ+ communities, not only the higher prevalence of risky substance use itself but also the earlier onset is of concern. Several issues are intersecting here with an earlier age of onset coinciding with one of the most critical developmental periods, adolescence, during which people form their identities, while also reporting strong levels of perceived discrimination and marginalisation due to their sexual orientation or gender identity. Chemsex has been subject to multiple studies over the past couple of years and can result in serious consequences including dependence, overdosing, hospitalisation and death. It is also related to an increase in ‘high-risk’ sexual activity and needle sharing, with those engaging in chemsex reporting a higher likelihood of being diagnosed with sexually transmissible infections and other blood-borne viruses, such as hepatitis C, than those not engaging in chemsex.

What do we understand about the reasons underlying these issues with substance use?

There is no single unique factor that can fully explain the disparities in substance use LGBTQ+ communities experience. Unfortunately, much of the discourse in this area is centred around the concept of suffering to explain substance use. However, substance use is not always the result of negative factors or experiences. Of course, some or even a lot of these disparities can be explained by looking at substance use as a mechanism to cope with discrimination, marginalisation and oppression, but there are a variety of factors involved with some more unique to LGBTQ+ communities, and others also affecting other communities. These include cultural factors, limited access to appropriate healthcare and community services or even targeted marketing. Chemsex is a great example, with LGBTQ+ communities and individuals showing generally very high levels of open-mindedness when it comes to sexuality and experimenting with sexual practices. This lower inhibition threshold makes it more likely they will also engage or experiment with risky practices such as chemsex. Research has shown that, while chemsex can be used as a coping mechanism, users report strong positive outcomes, such as enhanced sexual confidence, increased sex drive and sexual stamina and a more intense sexual experience in general.

What research is needed to further understand substance use and how it impacts the LGBTQ+ community?

The body of literature has developed a lot over the past ten years but more and different research is not only possible but also required. There is some evidence suggesting that the disparities in substance use are not equally distributed across LGBTQ+ populations, with some groups showing higher disparities than others. Research often still assumes that we are looking at a monolith block of identities when researching in LGBTQ+ communities. However, the reality is more complex than that and research needs to acknowledge and accommodate this reality. This means to ensure a good representation of different communities, particularly sex- and gender-diverse people and to look at these issues through an intersectional lens – that is, one that takes into account the interconnected nature of social categories and identities - as LGBTQ+ people do not exist in isolation from other groups. Furthermore, it is important to create more research with communities rather than about communities. Research too often focuses on aspects that are not a priority for LGBTQ+ communities. Community-driven research has the potential to be more valuable and useful to the lived experiences and structures of LGBTQ+ communities.

How does your own research further these goals?

My research is community-driven and community-informed. I consult with community organisations and community members about each idea I have, ensuring that my research is relevant and useful for them. To further my engagement, I volunteer for community organisations in my area and I’m involved as an active member. Being both a researcher in this area and a community member, this might be easier for me than for other researchers. My research also does not end with the publication of its results. I find it important to report my results back to the community, explain the findings, get their input into their interpretation, and assist them in using research findings to inform their services.

What else is important for our readers to understand about substance use in LGBTQ+ populations?

It is really important that we acknowledge the complexity of substance use and stop to pathologize the use of substances per se. The current lens used to understand, investigate and treat substance use and substance users is unlikely to reach those most in need of assistance. The public perception of substance use has changed dramatically over the past decade and research and practice need to ensure they are keeping up with community sentiments and cultures.

